# Pulsed Alternating Fields Magnetic Hyperthermia in Combination with Chemotherapy (5-Fluorouracil) as a Cancer Treatment for Glioblastoma Multiform: An In Vitro Study

**DOI:** 10.3390/nano15070556

**Published:** 2025-04-05

**Authors:** Lilia Souiade, Miguel-Ramon Rodriguez-Garcia, José-Javier Serrano-Olmedo, Milagros Ramos-Gómez

**Affiliations:** 1 Centro de Tecnología Biomédica, Universidad Politécnica de Madrid, Campus de Montegancedo, Pozuelo de Alarcón, 28223 Madrid, Spain; lilia.souiade@ctb.upm.es (L.S.); miguelramon.rodgarcia@ctb.upm.es (M.-R.R.-G.); josejavier.serrano@ctb.upm.es (J.-J.S.-O.); 2 Centro de Investigación Biomédica en Red para Bioingeniería, Biomateriales y Nanomedicina, Instituto de Salud Carlos III, 28029 Madrid, Spain; 3 Departamento de Tecnología Fotónica y Bioingeniería, ETSI Telecomunicaciones, Universidad Politécnica de Madrid, 28040 Madrid, Spain

**Keywords:** nanomedicine, magnetic hyperthermia, nanoparticles, pulsed AMFs, chemotherapy, cancer, CRT, ICD

## Abstract

Inducing magnetic hyperthermia (MHT) involves locally raising the temperature to 39–45 °C, which increases the susceptibility of tumor cells to therapeutic agents without damaging healthy tissues. Recent studies on trapezoidal pulsed alternating magnetic fields (TP-AMFs) have proven their considerable efficacy in increasing the temperature of magnetic nanoparticles (MNPs) compared to sinusoidal fields. Thermal therapies have been known to incorporate multiple combinations of therapeutic approaches to optimize the medical procedure for healing cancer patients such as chemotherapy and radiotherapy. The combination of MHT with chemotherapy aims to enhance the therapeutic effects against cancer due to the synergistic interaction in tumor cells. In this study, we aim to exploit the synergistic effects of combining MHT produced by TP-AMFs with a low concentration of 5-Fluorouracil (5-FU) to optimize the therapeutic outcomes in comparison to TP-AMFs MHT alone. Hence, we exposed a glioblastoma cell line (CT2A) incubated with iron oxide nanoparticles at 1 mg/mL to two cycles of MHT employing a trapezoidal-square waveform at 200 kHz and 2 mT for 30 min for each cycle, separated by a 45 min break, both as a single treatment and in combination with 0.1 μg/mL of 5-FU. Our findings demonstrated the efficacy of the synergistic effect between MHT treatment via TP-AMFs and the 5-FU, increasing the cell death to 58.9 ± 2%, compared to 31.4 ± 3% with MHT treatment alone. Cell death was primarily driven by the necrosis pathway (47.3 ± 2%) compared to apoptosis (11.6 ± 2). The addition of 5-FU enhanced the cytotoxic effect of MHT on CT2A cells, increasing the calreticulin (CRT) positive cells to 17 ± 1% compared to 10 ± 1% as produced by MHT treatment alone. Furthermore, this combination suggests that the employed treatment approach can promote immune system activation due to the exposure of CRT in the treated cells.

## 1. Introduction

**Figure 1:**
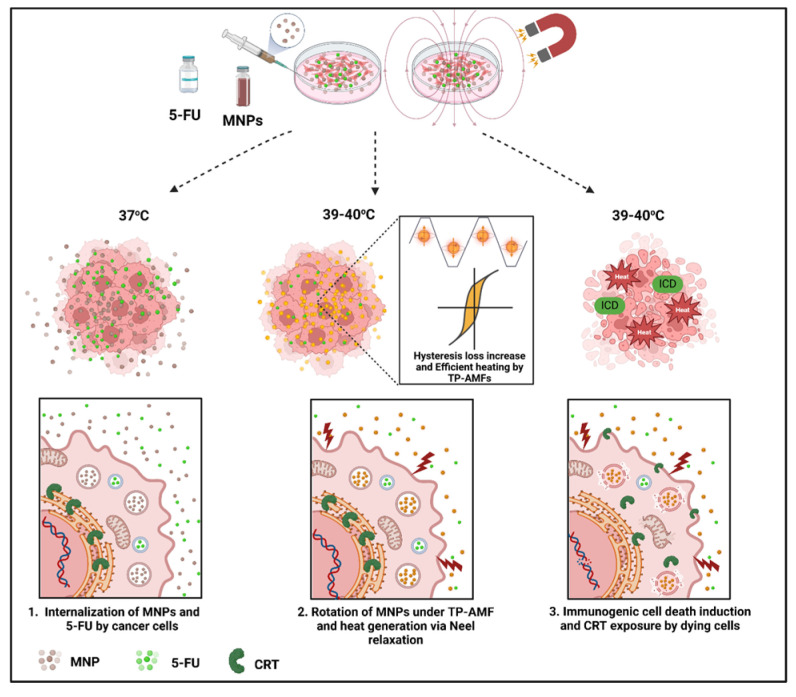
Schematic illustration of the synergistic effects between magnetic hyperthermia and chemotherapy in inducing immunogenic cell death. MNPs are efficiently internalized by cancer cells and their exposure to TP-AMFs allows an efficient heat release within cells causing permeabilization of biological membranes and enhancing the uptake of chemotherapeutic drugs (1, 2). The combinatory treatment of chemotherapy (5-FU) and MHT induces ER stress leading to CRT exposure, which could in turn trigger the activation of an immunogenic cell death (3).

Glioblastoma multiforme or GBM is the most common malignant brain tumor, traditionally treated with surgery, radiotherapy, and chemotherapy. Modern therapies include hyperthermia and magnetic hyperthermia technology, immunotherapy (cancer vaccines), virotherapy (oncolytic virus-induced immunogenic cell death), and gene therapy [1]. Hyperthermia (HT) is a cancer treatment that uses heat (39–45 °C) to damage cancer cells and inhibit tumor growth while minimizing harm to surrounding healthy tissues [2,3]. Magnetic nanoparticles (MNPs) are used in HT to target heat specifically towards the tumor tissues rather than healthy tissues, which is challenging in radiofrequency and microwave hyperthermia [4]. This modern oncological hyperthermic treatment is based on inducing heat through an external activation of iron oxide nano-mediators by an alternating magnetic field. Current advancements in MHT focus on active targeting through the improvement of the specificity of MNPs to target the tumoral area more effectively [5,6]. Moreover, the optimization of the magnetic field strength influenced by coil geometry, frequency and amplitude was also investigated to increase the efficacy of MHT treatment [7,8]. Recently, the application of trapezoidal pulsed alternating magnetic fields (TP-AMFs) instead of sinusoidal fields (SN-AMFs) had proven its efficacy in enhancing the efficiency of heating MNPs [9,10,11] and led to a considerable increase in cancer cell death within two different cell lines (CT2A and B16F10) compared to conventional treatment [12]. Furthermore, MHT has been applied in combination with conventional cancer therapies, such as chemotherapy, radiotherapy, gene therapy, and immunotherapy, to synergistically enhance therapeutic outcomes [13]. Chemotherapeutic drugs such as doxorubicin, paclitaxel, mitoxantrone, and oxaliplatin were considered as inducers of ICD due to their ability to eliminate tumor cells by provoking endoplasmic reticulum (ER) stress, leading to CRT release as seen in Figure 1, and heat shock protein 70 (HSP70) overexpression [14]. This ICD pathway plays a crucial role in stimulating an immune response to recognize and remove the remaining cancer cells [15,16]. Recent studies on human glioblastoma cells could elucidate the innate immunological effects of mild magnetic hyperthermia alone [17] and photothermal treatment combined with chemotherapy [18]. Numerous preclinical studies examined the application of MHT integrated with a chemotherapeutic drug (5-FU) for cancer treatment on different cancer cell lines and mouse models [19,20,21,22,23,24]. Our study focuses on enhancing the therapeutic effects of MNP-mediated MHT produced using a trapezoidal-square signal in combination with 5-FU for glioblastoma multiforme treatment (Figure 1).

## 2. Materials and Methods

### 2.1. Cell Culture

CT2A cancer cells were cultured in Dulbecco’s modified Eagle’s medium (DMEM) supplemented with 10% heat-inactivated Fetal Bovine Serum (FBS), 2 mM of glutamine, 100 units/mL antibiotics of penicillin, 100 µg/mL of streptomycin, and non-essential amino acids. All reagents were obtained from Thermo Fisher Scientific (Spain). Later, the cells were incubated in a humidified atmosphere with 5% CO_2_ at 37 °C.

### 2.2. XTT Assay for Examination of Cytotoxicity Effect

#### 2.2.1. 5-Fluorouracil

The 5-FU and dimethyl sulfoxide (DMSO) cytotoxicity in CT2A cells was determined using the XTT (2,3-bis-(2-methoxy-4-nitro-5-sulfophenyl)-2H-tetrazolium-5 carboxanilide) colorimetric assay. DMSO was tested since it is used as solvent for 5-FU, and it might be toxic for cells. Thus, CT2A cells were seeded in 24-well plates at a density of 3 × 10^4^ cells/cm^2^ and grown for 24 h.

Cells were then treated with 0.1, 0.125, and 0.2 μg/mL of 5-FU. For testing DMSO, cells were incubated with DMSO using the same amount present in each 5-FU concentration for 48 h, and the untreated cells were used as the control. The assay was repeated in triplicate. Later, the wells were washed twice and incubated with 350 µL of XTT master mix for an additional 1 h at 37 °C.

#### 2.2.2. Magnetic Nanoparticles

The 3-aminopropyl-trietoxysilane (APS)-coated positively charged superparamagnetic iron oxide nanospheres of 8.3 ± 2 nm were used in our study on a mouse glioblastoma cell line (CT2A cells). The synthesis procedure of APS-MNPs has been previously mentioned in Ref. [25]. Briefly, the synthesis was made by Massart’s coprecipitation protocol to prepare the maghemite nanosphere by mixing a 445 mL of FeCL_3_·6H_2_O and FeCL_2_·4H_2_O to 75 mL alkaline medium NH_4_OH (25%) under stirring at room temperature; then, a magnetic decantation was made using an appropriate magnet.

The precipitate was later washed three times, and a protocol of oxidation and activation of the nanoparticles’ surface proceeded, followed by an acidic treatment with 300 mL HNO_3_ under stirring. Next, the supernatant was separated by magnetic separation, and 75 mL of Fe (NO_3_)_3_ (1M) and 130 mL of water was added. The new mixture was heated up to boiling temperature and then cooled to room temperature. Later, the supernatant was removed, 300 mL of HNO_3_ was added, and the solution was washed many times using water. Finally, the coating of nanoparticles was realized using positively charged APS. To assess the cytotoxic effect of APS-MNPs, CT2A cells were cultured in P24-well plates (3 × 10^4^ cells/cm^2^) and allowed to grow for 24 h. Later, the nanoparticles were added at 1 mg/mL and incubated for 24 and 48 h. Cell viability was measured using the XTT assay kit. The cell viability was estimated by determining the absorbance of each well at 470 nm using a 96-well microplate reader, with the control absorbance value equated to 100%, and all other values were calculated relative to the control. Cell viability was calculated according to the following formula:

(1) $$Cell\;Viability=\frac{average\;absorbance\;of\;treated\;cells}{average\;absorbance\;of\;control\;cells}\times 100$$

### 2.3. Magnetic Hyperthermia Device

The pulsed alternating magnetic field used in this study is generated by a solenoid coil of a home-made heat induction device [10] which is able to produce a variety of waveforms which are trapezoidal-square (TS), trapezoidal (TP), trapezoidal-triangular (TT), and triangular (TR) plus the sinusoidal (SN) waveform and frequencies ranging from 100 kHz to 500 kHz and 1 MHz. The copper coil has 14 turns, an inner diameter of 6 cm, and an outer diameter of 7 cm. It surrounds a closed cooling system linked to a water bath to heat-up cancer cell samples to 37 °C.

### 2.4. Evaluation of Chemo(5-FU)-Magnetic Hyperthermia Therapy

To evaluate the increase of the therapeutic effect by combining chemotherapy with MHT against glioblastoma cells, in vitro experiments were performed under an AMF of 200 kHz and 2 mT, with 30 min of exposure for each hyperthermic cycle and 45 min of rest between the two exposures to allow the MHT system components (Transistors) and the solenoid coil to cool down and prevent any possible damage from their overheating. CT2A cancer cells were seeded in 35 mm cell culture dishes at 3×10^4^ cells/cm^2^ and cultured for 24 h. The cell culture dishes were divided into different experimental groups: the control group without any treatment, the group treated with magnetic field and chemotherapy (AMF + 5-FU), the group treated with iron oxide nanoparticles only (MNPs), the group treated with chemotherapy only (5-FU), the group treated with MHT alone (2 cycles of MHT), and the group treated with MHT plus chemotherapy (2 cycles MHT + 5-FU).

Once cells achieved 85% of confluence in culture dishes, APS-MNP were added at 1 mg/mL to the group treated with only MNPs and the group treated with chemo-magnetic hyperthermia, while 5-FU at 0.1 μg/mL was incubated with the group treated by only 5-FU, the group treated with AMF + 5-FU, and the group treated with chemo-magnetic hyperthermia. The in vitro treatment under AMF was initiated at 37 °C.

### 2.5. Annexin V/Propidium Iodide Assay (Apoptosis and Necrosis Quantification)

CT2A cell death was determined using Annexin V (ABCAM, Cambridge, UK) and Propidium Iodide (Sigma-Aldrich, St. Louis, MO, USA) for staining apoptotic and necrotic cells, respectively. Twenty-four hours after treatment, the cells were washed twice with PBS and once with 1× binding buffer. Then, the cells from each group were stained with Annexin V and PI for 15–20 min in the dark at room temperature, followed by Hoechst staining to visualize the nuclei. Annexin V is a green fluorescent protein that binds to the amino-phospholipid phosphatidylserine on the plasma membrane’s inner layer, which is externalized to the membrane’s outer layer during apoptosis, allowing the detection of apoptotic cells before they lose membrane integrity. Propidium iodide is a red fluorophore that marks the nucleus of necrotic cells. Hoechst is a cell-permeant fluorescent stain that selectively binds to DNA and marks the cell nucleus. The analysis of stained cells and image acquisitions were carried out using a fluorescence microscope (Leica DMI 3000B, Wetzlar, Germany). The excitation light wavelength for Annexin V, Hoechst, and PI was 649 nm, 350 nm, and 535 nm, respectively, while the emission light wavelengths were 670 nm, 461 nm, and 617 nm, respectively. The ImageJ software (version 1.53t, NIH, Bethesda, MD, USA) and GraphPad Prism software (version 8.01) were used to illustrate the results.

### 2.6. CRT Immunocytofluorescence Analysis

Immunocytofluorescence analysis was used to assess the CRT protein expression in CT2A cancer cells. Cell samples of the experimental groups (control, AMF, 5-FU, MNP, 2 cycles MHT, and 2 cycles MHT + 5-FU) were washed and fixed using 4% paraformaldehyde (PFA, Merck, Darmstadt, Germany) in 0.1 M phosphate buffer at pH 7.4 for 20 min at room temperature. Cells were then washed 3 times in PBS and blocked using 10% horse serum (Gibco/Life Technologies, Carlsbad, CA, USA) for 30 min. Later, the cells were incubated overnight at 4 °C in the dark with an anti-CRT antibody diluted to 1:400. Finally, the samples were rinsed and incubated with anti-FITC secondary antibody diluted to 1:400 in PBS for 1 h at room temperature. Fluorescence microscopy images were taken, and the total number of cells in each field was determined by counting the nuclei stained with Hoechst, while CRT-positive cells were determined by counting the cells stained with an anti-CRT antibody in green. All image analyses were performed using ImageJ software (version 1.53t, NIH, USA). The percentage of CRT-positive cells was then calculated using the following formula:

(2) $$CRT\;positive\;cells\;(\%)=\frac{Cells\;stained\;in\;green\;(CRT)}{Total\;number\;of\;cells}\times 100$$

### 2.7. Statistical Analysis

The results obtained are shown as the mean ± SEM from three experiments performed in triplicate and were examined statistically with GraphPad Prism Version 8.0.1 for Windows. The data were evaluated for normality using the Shapiro–Wilk test and presented as the mean ± SEM. The ANOVA test was used to quantify differences between data groups, with statistical significance defined as *p* < 0.05.

## 3. Results

### 3.1. In Vitro Cytotoxicity Evaluation

**Figure 2:**
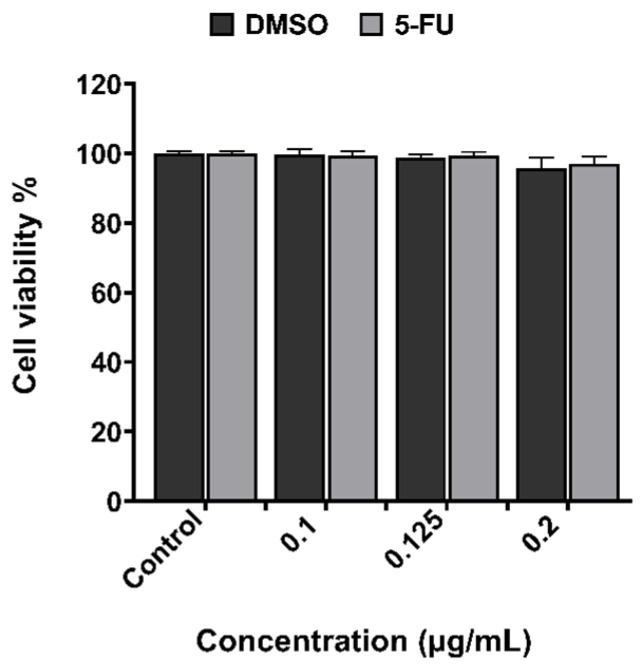
Quantification of cytotoxicity performed by the XTT assay of 5-FU and its solvent (DMSO) in CT2A cells 48 h after incubation with different concentrations. Data are represented as mean ± SEM (*n* = 3).

**Figure 3:**
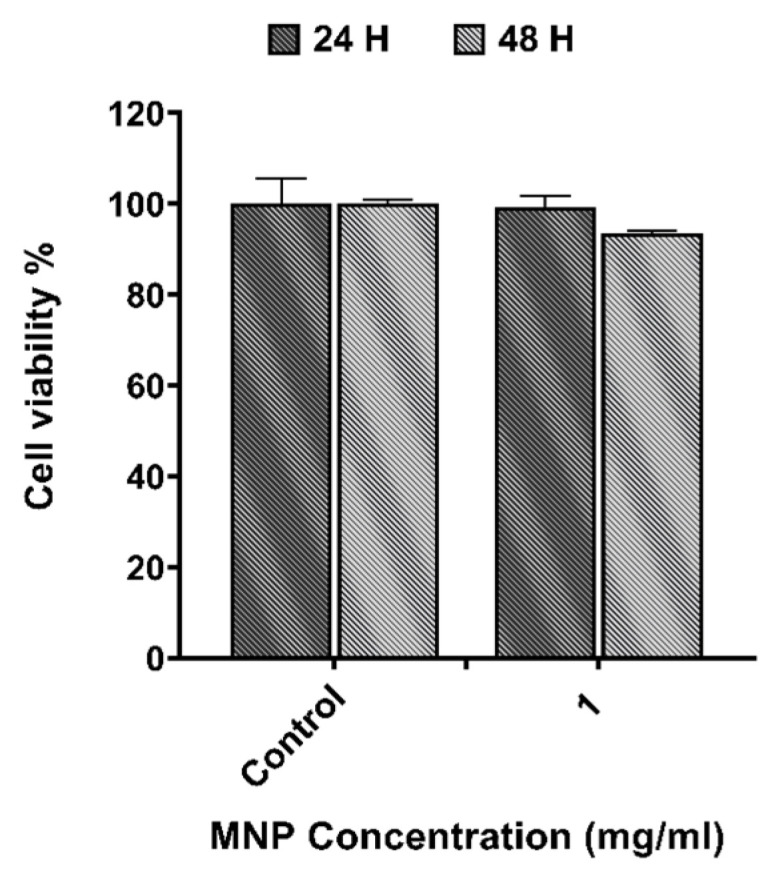
Cell viability of CT2A cancer cells incubated with APS-MNPs at 1 mg/mL for 24 h and 48 h revealed by XTT assay. Data are represented as mean ± SEM (*n* = 3).

The cytotoxicity results obtained from the XTT assay for 5-FU at different concentrations, and for APS-MNPs using 1 mg/mL after 24 h and 48 h in CT2A cells, are depicted in Figure 2 and Figure 3. The DMSO toxicity was evaluated in parallel because it serves as a solvent for the preparation of the chemotherapeutic drug. The results showed non-significant cytotoxicity on CT2A cells 48 h after incubation with 5-FU and DMSO at different concentrations of 0.1 μg/mL, 0.125 μg/mL, and 0.2 μg/mL (Figure 2). The lowest 5-FU concentration (0.1 μg/mL) was chosen for the combined therapy since this concentration did not induce any decrease in cell viability. On the other hand, APS-MNPs showed no cytotoxic effect on cell viability after 24 h of incubation. After 48 h of incubation, a negligible decrease in viability to 93% was found in CT2A cells (Figure 3). Based on previous results [12], APS-MNPs were used at 1 mg/mL since no evident cell cytotoxicity was observed at this concentration.

### 3.2. In Vitro Chemo(5-FU)-Magnetic Hyperthermia Therapy on Glioblastoma Cell Line

**Figure 4:**
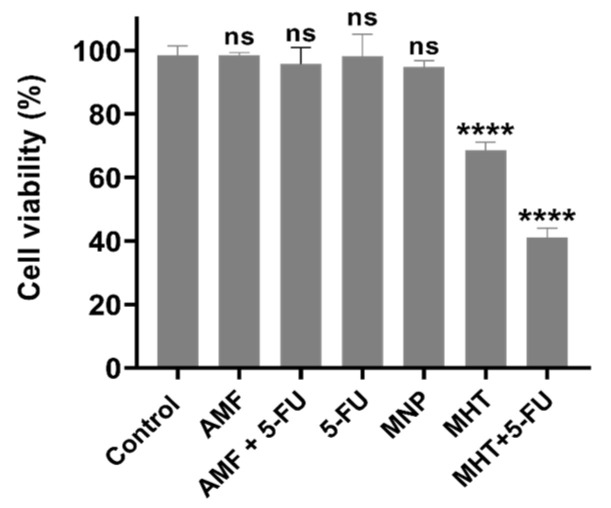
Cell viability assessment with fluorescence microscopy 24 h after MHT treatment induced by TP-AMFs at 200 kHz/2 mT and 1 mg/mL of APS-MNP in combination with 0.1 μg/mL of 5-FU to provoke cell death in CT2A cells groups (Control, AMF, AMF + 5-FU, MNP, MHT, and MHT + 5-FU). Data are represented as mean ± SEM (*n* = 3). Not significant (ns). **** *p* < 0.0001 versus control.

The results obtained on CT2A cells after MHT treatments generated by TS signal combined with the chemotherapeutic drug 5-FU are illustrated in Figure 4. To examine the in vitro therapeutic potential of different treatments and to determine the cell death type (apoptosis or necrosis) we prepared different predefined experimental groups of CT2A cell cultures: Control, AMF, 5-FU, MNP, MHT only (two cycles), and MHT + 5-FU (two cycles). Annexin V and PI analysis was performed 24 h after treatment of CT2A cells preincubated with APS-MNP (1 mg/mL) and 5-FU (0.1 μg/mL) for 24 h followed by exposure to two MHT cycles (30 min each cycle and 45 min time of rest between cycles) using a TS waveform at 200 kHz of frequency and 2 mT of amplitude. The APS-MNPs were chosen for this study due to their small size and positively charged coating, which enables their rapid internalization by cells, since cells have a net negative charge on their outer surface.

Regarding the numerical data of the quantified cell viability using fluorescence microscopy, as shown in Figure 4, the treatment with MHT alone resulted in a significant decrease in cell viability of 31.4 ± 3% in CT2A cells. In contrast, chemo-magnetic hyperthermia treatment led to a further significant decrease of 58.9 ± 2% in CT2A cell viability compared to the untreated control group. Treatment with chemotherapy alone did not produce a significant reduction in viability, with or without AMF.

**Figure 5:**
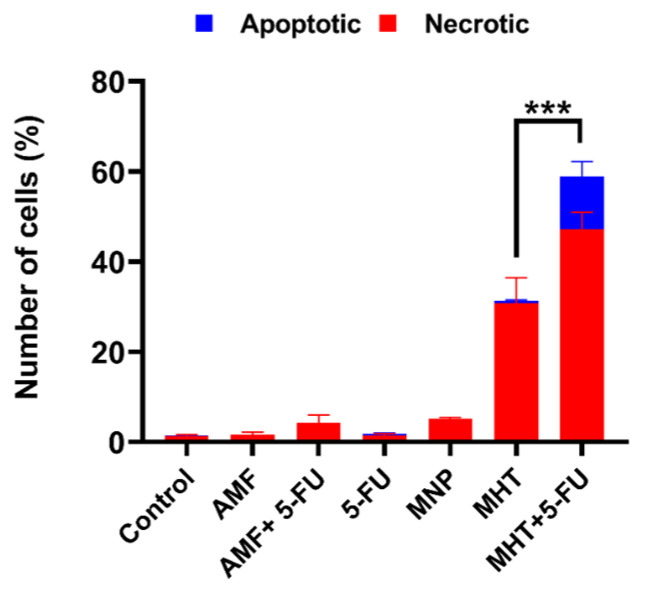
Analysis of cell death pathway using Annexin V/PI in CT2A treated and untreated groups to determine apoptosis and necrosis relative percentage. Data are represented as mean ± SEM (*n* = 3); *** *p* < 0.001 versus control.

**Figure 6:**
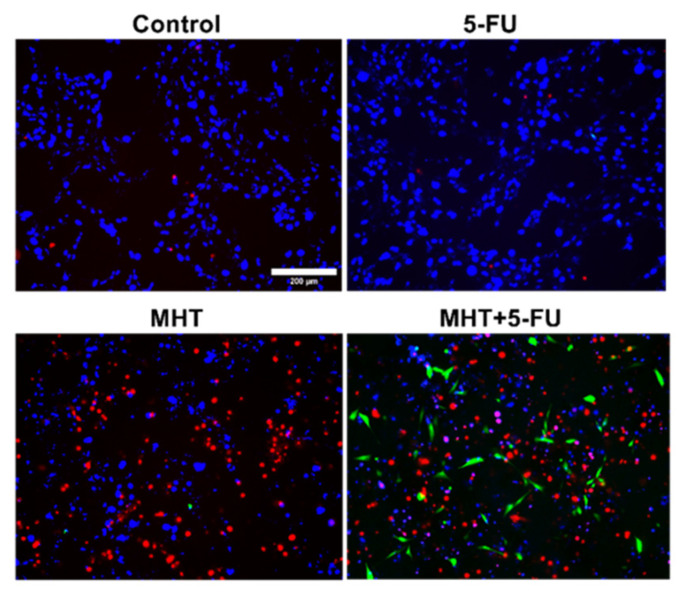
Fluorescence microscopy images of CT2A cells obtained after Annexin/PI staining show the group treated with chemotherapy alone (5-FU), the group treated with two consecutive cycles of MHT using a TS waveform at 200 kHz and 2 mT (MHT), and the group treated with two consecutive cycles of MHT combined with 5-FU at 0.1 μg/mL (MHT + 5-FU), compared to the control group. In the images, apoptotic cells are observed in green (Annexin V), necrotic cells in red (PI), and the nuclei of all cells in blue (Hoechst). Scale bar: 200 μm.

The analysis of cancer cell death in Figure 5, using Annexin/PI staining, showed that cell death was significantly higher in the group treated with MHT and chemotherapy (47.3 ± 2%) compared to the group treated with MHT alone (30.8 ± 3%). The other tested groups did not show significant cell death. Additionally, the combination of MHT and chemotherapy resulted in a significant increase in the number of apoptotic cells (11.6 ±2%) compared to the MHT-treated group, where apoptotic cells were barely observed. The fluorescence microscopy images in Figure 6 of the cell groups exposed to chemotherapy (5-FU) alone, MHT alone, and MHT + chemotherapy (5-FU) and the control group illustrate further the effective therapeutic impacts of the combined treatment against CT2A cells.

**Figure 7:**
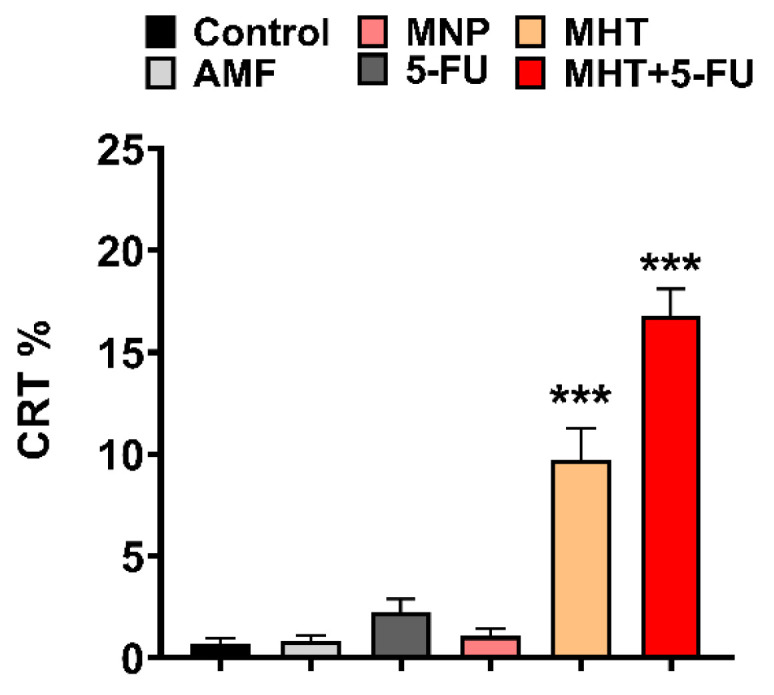
Quantification data of CRT-**positive** cells in the CT2A cell line 24 h after treatment, calculated as the percentage of cells positive for the CRT marker over the total number of cells. Data are represented as mean ± SEM (*n* = 3); *** *p* < 0.001 versus control. Scale bar: 200 µm.

**Figure 8:**
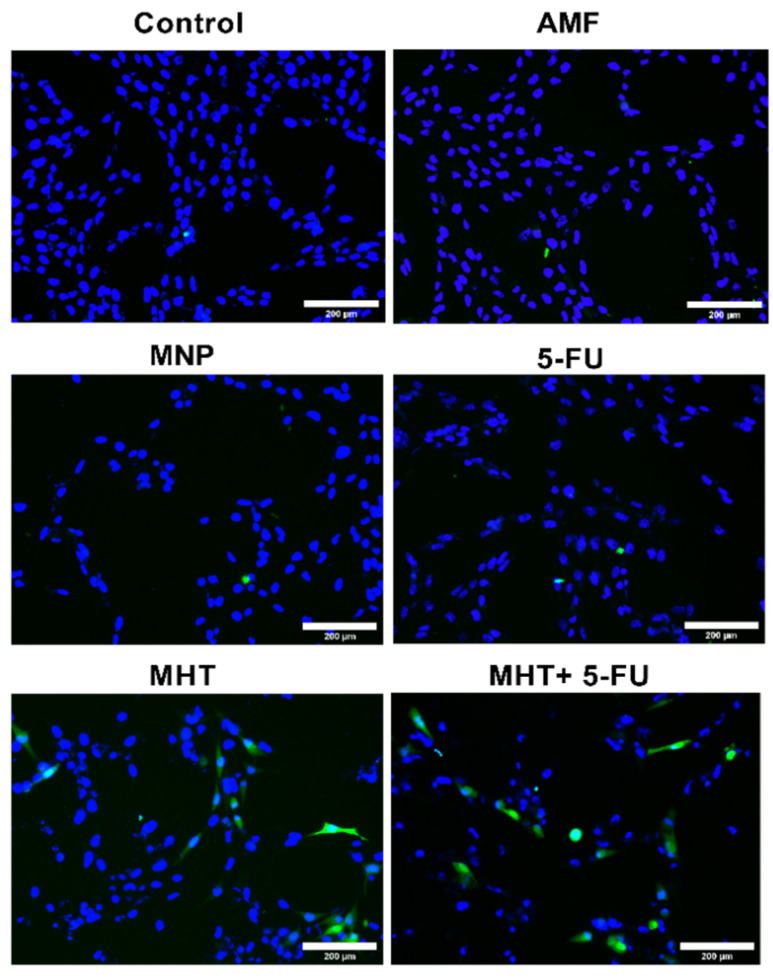
Immunocytofluorescent images of the ICD-associated marker (CRT) in CT2A cells 24 h after treatment. The representative images correspond to the groups exposed to the following treatment: AMF, MNP, 5-FU, MHT, and MHT + 5-FU, compared to the untreated group.

Furthermore, another type of cell death, known as immunogenic cell death (ICD), can be identified through the activation of certain damage-associated molecular patterns (DAMPs) in dying cancer cells following MHT and 5-FU treatments. This ICD contributes to the recognition of dying cells by APCs. DAMPs include the overexpression and translocation of calreticulin (CRT), heat shock proteins (HSP70 and HSP90), extracellular release of adenosine triphosphate (ATP), high-mobility group box-1 (HMGB1), type I IFNs, and members of the IL-1 cytokine family [14,26]. Hence, to analyze whether a specific treatment enhanced ICD in CT2A cells, we evaluated the number of CRT-positive cells after the different treatments. CRT serves as a danger signal that triggers ICD, activating systemic antitumor immunity. Differences among the experimental groups were determined based on the rate of CRT-positive cells quantified from fluorescence microscopy images obtained after an immunostaining assay using an anti-CRT antibody (Figure 7 and Figure 8).

The data in Figure 7 show that CRT expression in CT2A cells significantly increased in the group with MHT treatment (two cycles of MHT) to 10 ± 1% and in the group receiving the combined treatment (two of cycles MHT + 5-FU) to 17 ± 1%, compared to the control group. The other experimental groups exposed to AMF, 5-FU, and MNPs did not show significant CRT expression. Therefore, these data suggest that the combination of MHT and 5-FU treatments increases the production of CRT-positive cells, resulting in a more effective method for eliminating cancer cells, as this type of cell death can enhance the immune response of the organism to eradicate cancer cells.

## 4. Discussion

The combination of hyperthermia with chemotherapy represents a new approach that has demonstrated numerous advantages in several studies. In Ref. [27], an in vivo study was conducted to eliminate GBM cells using drug-loaded lipid MNPs for combined hyperthermia and chemotherapy, proving to be an effective local treatment. Similarly, the studies in Refs. [20,23] observed a substantial tumor remission in a mouse model after treatment with hyperthermia and 5-FU, compared to hyperthermia or chemotherapy alone. The novelty of our work lies in the combination of MHT generated by a non-sinusoidal signal with 5-FU at a low concentration in order to achieve a substantial optimization in cancer treatment and promoting an immune response against tumor cells. Various studies have demonstrated the potential that is offered by the pulsed alternating magnetic fields to enhance MHT over the sinusoidal fields. In our previous report [12], we demonstrated the efficacy of the non-sinusoidal waveforms in improving MHT treatment efficiency against two cancer cell lines, glioblastoma and melanoma, compared to traditional treatment. More specifically, we found that the TS waveform, selected for the present study, was the most efficient type of waveform in inducing cancer cell death. Furthermore, a developed pulsed magnetic field (PMF) generator has shown fourfold higher efficiency compared to sinusoidal waves [28].

The treatment strategy employed in this study was designed to optimize the therapeutic potential against the GBM cell line by MHT treatment combined with chemotherapy at a low concentration (Figure 5) and inducing the ICD pathway (Figure 7). Thus, the chemotherapeutic drug was utilized at the lowest non-cytotoxic concentration (0.1 μg/mL) with the biocompatible APS-MNPs (1 mg/mL) (Figure 2 and Figure 3, respectively). The induction of ICD leads to increased expression of DAMPs, such as CRT, by dying cells, which in turn evoke an adaptive immune response [16,29]. The CRT proteins serve as an “eat me” signal for immune cells, and this is critical for ICD induction, causing dying cells to become immunostimulatory entities. Once CRT binds to APCs via specific receptors, leading to their stimulation, it then promotes APCs to further activate the adaptive immune response, including the activation of Naive T cells, along with CD4 [17]. Eventually, the quantification of cell death by annexin/PI staining assay among the experimental groups was consistent with the immunocytofluorescent analysis of CRT-positive cells. The TP-AMFs magnetic hyperthermia treatment combined with 5-FU led to considerable cell death (58.9 ± 2%) compared to MHT treatment alone (31.4 ± 3%) (Figure 5) and to intense ICD as evidenced by a higher CRT staining compared to MHT or chemotherapy alone (Figure 7). This suggests the translocation of CRT to the membrane surface of damaged cells [17]. MHT treatment alone, using two cycles of exposure to AMF for 30 min each cycle separated by a 45 min break, resulted in a cell mortality rate of 31 ± 3%, compared to the cell death rate of 24 ± 3% obtained with one cycle [12]. In Ref. [17], three consecutive cycles of MHT by a conventional sinusoidal waveform have been applied at 182 kHz and 16 mT, with 30 min of exposition to SN-AMF in each cycle separated by 5 min break, and have been shown to cause a reduction in cell viability by 30% in human GBM cells 24 h post-treatment. Furthermore, the combination of TP-AMFs magnetic hyperthermia and chemotherapy resulted in a significantly higher cell mortality rate of 58.9 ± 2%, driven 47.3 ± 2% through necrosis and 11.6 ± 2% through apoptosis. This demonstrates the ability of low-intensity TP-AMFs magnetic hyperthermia in improving cancer treatment when combined with traditional approaches. Interestingly, our findings revealed that necrotic cell death induced by chemo-magnetic hyperthermia was significantly higher than that observed in the group of cells treated with MHT alone. Moreover, apoptotic cells were only present in the group treated with chemo-magnetic hyperthermia. These results may be attributed to the ability of MHT treatments to enhance the effectiveness of chemotherapy by sensitizing cancer cells to chemotherapeutic drugs and facilitating their uptake into cells by increasing the cell membrane fluidity [30], ultimately leading to greater damage to cancer cells. The results obtained further support the approach of combining different cancer treatments to induce various types of cell death, such as ICD and apoptosis. These mechanisms are particularly relevant in cancer therapy because, unlike necrosis, they do not cause inflammation or damage to nearby healthy tissues [31].

## 5. Conclusions

Magnetic hyperthermia using biocompatible iron oxide nanoparticles of small size, which can easily cross biological barriers to target tumor tissues, along with non-harmonic waveforms instead of harmonic ones, offers considerable efficiency in eliminating CT2A cells. The use of TP-AMFs for anticancer therapy provides a substantial advantage by allowing treatments at low magnetic field intensities with more effective signal waveforms.

In this study, we demonstrate that the combination of MNP-based MHT with chemotherapeutic drugs creates a significant therapeutic improvement for eliminating cancer cells. In summary, we showed the excellent therapeutic efficacy of TP-AMFs applied for two consecutive cycles in combination with 5-FU for eliminating CT2A GBM cells. Additionally, this combined therapy produced enhanced apoptotic cell death and an increased expression of CRT, a DAMP molecule that promotes the recognition of dying cells by the immune system. This suggests that the combination of chemotherapy with MHT could potentially activate the immune system to target and eliminate the remaining cancer cells. These outcomes highlight the synergistic effects between TP-AMFs magnetic hyperthermia and chemotherapy, resulting in significant and irreversible damage, as well as triggering the ICD pathway, compared to MHT or chemotherapy alone.

## Data Availability

The presented study data are available on request from the corresponding author.

## Abbreviations

MHT	Magnetic hyperthermia
MNPs	Magnetic nanoparticles
TP-PAMFs	Trapezoidal pulsed alternating magnetic fields
5-FU	5-Fluorouracil
ICD	Immunogenic cell death
GBM	Glioblastoma multiform
HT	Hyperthermia
SN	Sinusoidal
TS	Trapezoidal-square
TT	Trapezoidal-triangular
TR	Triangular
ER	Endoplasmic reticulum
CRT	Calreticulin
DMEM	Dulbecco’s modified eagle’s medium
FBS	Fetal bovine serum
APS	3-aminopropyl-trietoxysilane
ROS	Reactive oxygen species
PFA	Paraformaldehyde
SEM	Standard error of the mean
HSP	Heat shock protein
DAMPs	Damage associated molecular patterns
APCs	Antigen presenting cells
ATP	Adenosine triphosphate
HMGB1	High-mobility group box-1
PI	Propidium Iodide
